# Tumor volume is more reliable to predict nodal metastasis in non-small cell lung cancer of 3.0 cm or less in the greatest tumor diameter

**DOI:** 10.1186/s12957-020-01946-0

**Published:** 2020-07-15

**Authors:** Bei Jia, Biao Chen, Hao Long, Tiehua Rong, Xiaodong Su

**Affiliations:** 1 Department of Thoracic Surgery, Sun Yat Sen University Cancer Center, Guangzhou, China; 2 State Key Laboratory of Oncology in Southern China and Collaborative Innovation Center for Cancer Medicine, Guangzhou, China; 3 Lung Cancer Institute, Sun Yat Sen University, Guangzhou, China

**Keywords:** Non-small cell lung cancer, Tumor volume, Greatest tumor diameter, Lymph node metastases, Pulmonary nodule, Surgical resection

## Abstract

**Background:**

In this study, we sought to evaluate the correlation between TV, GTD, and lymph node metastases in NSCLC patients with tumors of GTD ≤ 3.0 cm.

**Methods:**

We retrospectively analyzed the characteristics of clinicopathologic variables for lymph node involvement in 285 NSCLC patients with tumors of GTD ≤ 3.0 cm who accepted curative surgical resection. The TVs were semi-automatically measured by a software, and optimal cutoff points were obtained using the X-tile software. The relationship between GTD and TV were described using non-linear regression. The correlation between GTD, TV, and N stages was analyzed using the Pearson correlation coefficient. The one-way ANOVA was used to compare the GTD and TV of different lymph node stage groups.

**Results:**

The relationship between GTD and TV accorded with the exponential growth model: *y* = 0.113e^1.455x^ (*y* = TV, *x* = GTD). TV for patients with node metastases (4.78 cm^3^) was significantly greater than those without metastases (3.57 cm^3^) (*P* < 0.001). However, there were no obvious GTD differences in cases with or without lymph node metastases (*P* = 0.054). We divided all cases into three TV groups using the two cutoff values (0.9 cm^3^ and 3.9 cm^3^), and there was an obvious difference in the lymphatic involvement rate between the groups (*P* < 0.001). The tendency to metastasize was greater with higher TV especially when the TV was > 0.9–14.2 cm^3^ (*P* = 0.010).

**Conclusions:**

For NSCLC tumors with GTD ≤ 3.0 cm, TV is a more sensitive marker than GTD in predicting the positive lymph node metastases. The likelihood for metastasis increases with an increasing TV especially when GTD is > 2.0–3.0 cm.

## Background

Lung cancer remains one of the most common type of malignant tumors in the world, with over 1.5 million new cases each year [1]. Among all lung cancer patients, non-small cell lung cancer (NSCLC) occupies approximately 80% [2]. Despite the improvements of medical methods in surgical resection, radiotherapy, or chemotherapy, the prognosis of NSCLC patients is still not satisfactory [3, 4].

Based on the definition of the Union for International Cancer Control (UICC)/American Joint Committee on Cancer (AJCC), the tumor, node, and metastasis (TNM) stage is the single most significant guideline for choosing the treatment methods and predicting the prognosis for NSCLC patients [5–7]. In 2017, the latest 8th edition TNM staging system was published in an effort to improve prognostic accuracy for NSCLC patients at each stage. According to this criteria, both the greatest tumor diameter (GTD) and lymph nodes remain the primary descriptive prognostic factors for NSCLC patients [8]. The relationship between GTD and lymph node metastases in clinical staging and choosing treatment methods accurately has been discussed in many previous studies [9–20]. Some studies revealed that there exists a close relationship between these two factors in that GTD can be used to predict positive lymph node metastases [10, 11, 15, 19, 20]. However, others concluded that there is no such specific correlation between these two factors [13, 16, 18]. The main reason for these different results is due to the discrepancy in the irregular shape of the tumor mass, even those with the same sizes [21]. In other words, GTD does not sufficiently reflect the true tumor burden, especially for pulmonary nodules (GTD ≤ 3.0 cm). Therefore, a more reliable indicator other than GTD is desired to predict lymph node metastases.

In recent years, computed tomography (CT) has become the standard procedure for non-invasive staging, treatment response evaluation, and prognosis prediction for NSCLC patients [22]. Thanks to the advanced imaging technology, more and more pulmonary nodules have been increasingly identified [23]. Therefore, the clinical characteristics in patients with pulmonary nodule lung NSCLCs including lymph node metastases and tumor size have been of great interest [13–15, 17, 19, 24].

The tumor volume (TV) can be easily calculated and collected with the assistance of the CT software [25, 26]. Since TV has been proven to reflect the tumor burden more accurately, it has been suggested to have a greater impact on the prognosis of NSCLC patients than tumor size does [27, 28]. Several previous studies demonstrated that TV is an independent adverse prognostic factor for post-surgery NSCLC patients [29, 30]. However, for NSCLCs of GTD ≤ 3.0 cm, the correlation between TV and lymph node metastases has not been well established, and the comparison between GTD and TV and their correlation with positive lymph node metastases also needs to be further studied.

In this study, we aimed to evaluate whether TV is a better factor for predicting the lymph node metastases than GTD and its clinical significance in NSCLCs patients with tumors of GTD ≤ 3.0 cm.

## Methods

### Patients

This study was approved by the ethics committee of the Sun Yat-Sen University Cancer Center and in accordance with the principles laid down in the Declaration of Helsinki. We reviewed patients diagnosed with NSCLC in our center from January 1, 2010, to December 31, 2012, retrospectively. If they did not undergo curative surgical resection (lobectomy or bilobectomy), had tumors with GTD > 3.0 cm, were identified with other types of cancer or multiple tumors, accepted neo-adjuvant chemotherapy, had no imaging records in our center or had an unmeasurable tumor on CT, or for other reasons they were excluded from our data. The patients’ characteristics were obtained through a standardized medical data collection form based on the 8th edition TNM classification. The pathological examination of tumors was carried out by the experienced pathologists in our center.

The preoperative examinations consisted of regular physical examination, chest X-rays, contrast-enhanced chest and upper abdomen CT scan, brain magnetic resonance imaging (MRI), radionuclide bone scans, pulmonary function test, bronchoscopy, electrocardiography, and blood tests.

Lung resection was performed through thoracotomy (open) or video-assisted thoracoscopic surgery (VATS). The extent of resection included lobectomy, bilobectomy, and pneumonectomy. Mediastinal lymph node dissection was done by surgeons after lung resection routinely.

### Follow-up

All patients in our study were followed up at the outpatient clinic in our center. For the first 2 years after surgery, the follow-up was every 3 to 4 months, then, every 6 months for 3 to 5 years and then, once a year. Standard procedures included physical examination, chest X-rays, abdominal and cervical ultrasonography, and chest as well as abdominal CT scans (once a year). Overall survival (OS) was calculated as the time from the surgery date to the death date or the last follow-up.

### TV and GTD measurement

The CT images (PHILIP Ict) were reviewed independently by an experienced radiologist without the background information of patients. CT images were viewed on standard lung windows (level − 500 HU; width 1500 HU) with section thickness of 1.0 mm. The TV and GTD were measured semi-automatically using the PHILIPS IntelliSpace Portal v5.0.2.40009 software (Philips Healthcare, Eindhoven, The Netherlands) (Supplemental Figure 1).

### Statistical analysis

The Chi-squared test was applied to analyze the characteristics of clinicopathologic variables for each lymph node stage. The relationship between GTD and TV were described by non-linear regression, which was tested with the *F* test. *T* test was used to test the coefficients and constants of this model. The optimal cutoff points of TV were determined using the X-tile Software version 3.6.1 (Yale University School of Medicine, New Haven, CT, USA). The Kaplan-Meier survival analysis was conducted to compare the OS of each TV groups. The Pearson correlation coefficient was used to analyze the correlation between GTD, TV, and N stage. The one-way ANOVA was used to compare GTD and TV between groups of cases with or without metastatic lymph nodes. The statistical analysis was performed using Statistical package for social sciences (SPSS) version 23.0.0.0 for Mac system (SPSS Inc., Chicago, IL, USA). A significant difference was affirmed when the *P* value (two-tailed test) was less than 0.05.

## Results

**Fig. 1:**
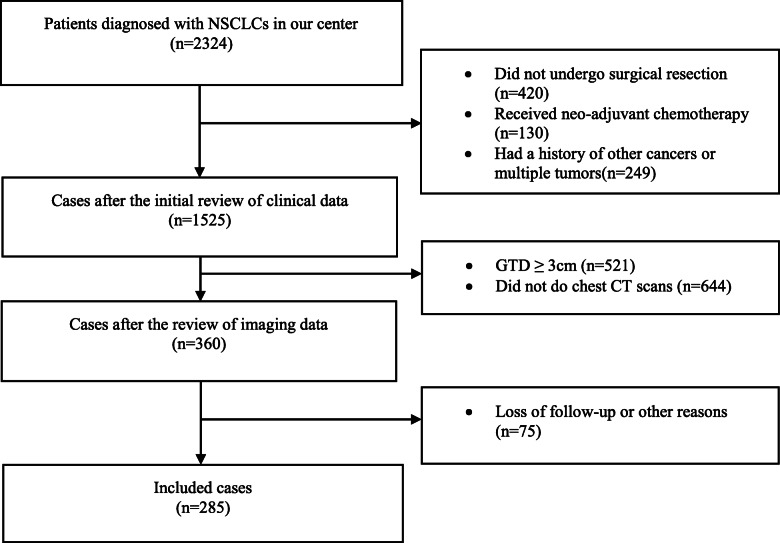
The flow chart of case collection and screening

A total of 2324 patients diagnosed with NSCLC were enrolled in the study. After exclusion for several reasons as shown in the case collection and screening flow chart in Fig. 1, 285 patients were included in the study.

### Patients’ characteristics

The patients’ characteristics with each N stage are summarized in Table 1. The number of N0, N1, and N2 cases was 204 (71.6%), 30 (10.5%), and 51 (17.9), respectively. The results showed that histology type (*P =* 0.038) and histologic differentiation (*P* < 0.001) were associated with different N stages.

**Table 1:** The relationship between clinicopathologic characteristics and lymph node involvement

	Cases (%)	pN0 (%)	pN1(%)	pN2 (%)	***P*** value
Total	285 (100)	204 (71.6)	30 (10.5)	51 (17.9)	
Sex					0.948
Male	155 (54.4)	111 (38.9)	17 (6.0)	27 (9.5)	
Female	130 (45.6)	93 (32.6)	13 (4.6)	24 (8.4)	
Age					0.812
< 70	240 (84.2)	170 (59.6)	26 (9.1)	44 (15.4)	
≥ 70	45 (15.8)	34 (11.9)	4 (1.4)	7 (2.6)	
Smoking					0.981
No	166 (58.2)	119 (41.8)	17 (6.0)	30 (10.5)	
Yes	119 (41.8)	85 (29.8)	13 (4.6)	21 (7.3)	
Type of surgery					0.199
Open	170 (59.6)	115 (40.4)	20 (7.0)	35 (12.3)	
VATS	115 (40.4)	89 (31.2)	10 (3.5)	16 (5.6)	
Excision site					0.364
Lobectomy	280 (98.2)	201 (70.5)	30 (10.5)	49 (17.2)	
Pneumonectomy	5 (0.8)	3 (1.1)	0 (0)	2 (0.7)	
Tumor location					0.967
Right upper	79 (27.7)	56 (19.6)	8 (2.8)	15 (5.3)	
Right median	26 (9.1)	21 (7.4)	2 (0.7)	3 (1.1)	
Right lower	46 (16.2)	33 (11.6)	5 (1.8)	8 (2.8)	
Left upper	77 (27.0)	54 (18.9)	9 (3.1)	14 (4.7)	
Left lower	40 (14.0)	28 (9.8)	5 (1.8)	7 (2.6)	
Others	17 (6.0)	12 (4.2)	1 (0.4)	1 (0.4)	
Histology					0.038
Adenocarcinoma	232 (81.4)	163 (57.2)	26 (9.1)	43 (15.1)	
Squamous cell carcinoma	35 (12.3)	28 (9.8)	4 (1.4)	3 (1.1)	
Others	18 (6.3)	13 (4.6)	0 (0)	5 (1.7)	
Histologic differentiation					< 0.001
Poorly differentiated	96 (33.7)	57 (20)	8 (2.8)	31 (10.9)	
Moderately differentiated	153 (53.7)	13 (4.6)	21 (7.4)	19 (6.7)	
Well differentiated	36 (12.6)	34 (11.9)	1 (0.4)	1 (0.3)	
Visceral pleural invasion					0.752
Absent	167 (58.6)	120 (42.1)	19 (6.7)	28 (9.8)	
Present	118 (41.4)	84 (29.5)	11 (3.9)	23 (8.0)	

### Correlation between GTD and TV

**Fig. 2:**
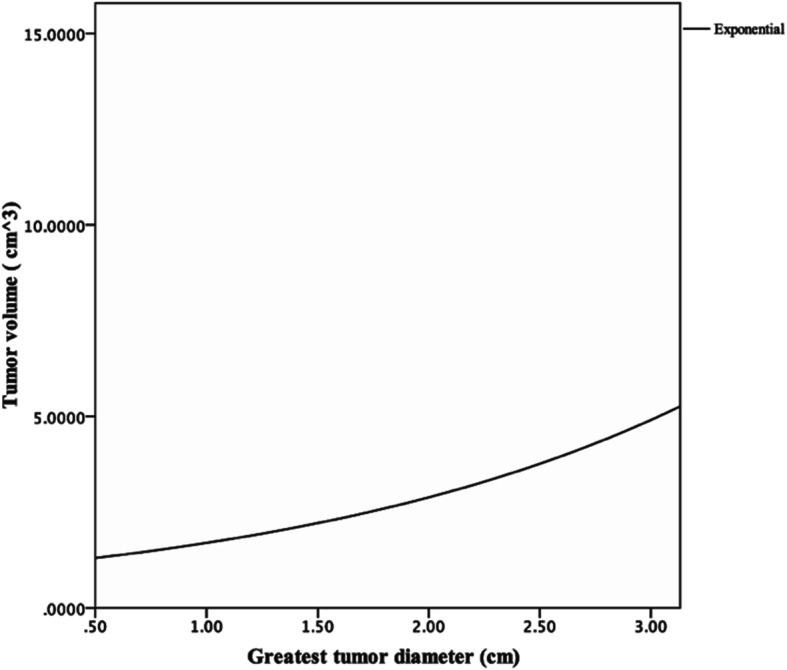
The exponential growth model curve between GTD and TV. The relationship between GTD (cm) and TV (cm^3^) is in accordance with the exponential growth model: *y* = 0.113e^1.455x^ (*y* = TV, *x* = GTD)

The correlation analysis revealed a proportional correlation between GTD and TV with the exponential growth model whose equation was *y* = 0.113e^1.455x^ (*y* = TV, *x* = GTD) (Fig. 2).

### GTD and TV in patients with or without metastatic lymph nodes

Unlike GTD (*P* = 0.054), TV for patients with node metastases (4.78 cm^3^, 95% CI 4.15–5.40 cm^3^) was significantly greater for those without metastases (3.57 cm^3^, 95% CI 3.21–3.94 cm^3^) (*P* = 0.001) (Table 2).

**Table 2:** The GTD and TV in patients with or without metastatic lymph node metastases

Lymph node metastases	Total (95% CI)	Negative (95% CI)	Positive (95% CI)	***P*** value
TV mean (cm^3^)	3.92 (3.60–4.23)	3.57 (3.21–3.94)	4.78 (4.15–5.40)	0.001
GTD mean (cm)	2.22 (2.16–2.29)	2.17 (2.09–2.49)	2.35 (2.24–2.47)	0.054

### TV optimal cutoff value

According to the overall survival (OS) of N0 patients, we acquired two optimal cutoff values using the X-tile software: 0.9 cm^3^ and 3.9 cm^3^. According to the equation of GTD and TV mentioned above, 0.9 cm^3^ and 3.9 cm^3^ were corresponded to 1.43 cm and 2.43 cm of GTD. The range of TV was from 0.2 to 14.2 cm^3^. Then, we divided all cases into three TV groups: V1 ≤ 0.9 cm^3^, V2> 0.9–3.9 cm^3^, V3 > 3.9–14.2 cm^3^. The OS of V1, V2, and V3 groups for N0 cases was 86.7%, 81.0%, and 75.7% respectively (*P* = 0.0375) (Table 3).

**Table 3:** The OS of V1, V2, and V3 groups for N0 cases

TV groups	Cases	OS (%)	***P*** value
Total	204	79.9	0.0375
V1 ≤ 0.9 cm^3^	30	86.7	
V2 0.9–3.9 cm^3^	100	81.0	
V3 3.9–14.2 cm^3^	74	75.7	

### Characteristic analysis of metastatic lymph nodes in patients by GTD and TV groups

The positive lymph node metastases rates of V1, V2, and V3 groups were 15.8%, 22.2%, and 38.8%, respectively (*P* = 0.004). In the metastatic cases, further analysis showed that the rate of N2 of each TV group was 50%, 57.1%, and 68.1%, respectively (*P* = 0.002).

For the purpose of comparing with TV, we also divided all cases into three GTD groups using the TNM staging classification standard: G1 ≤ 1.0 cm, G2> 1.0–2.0 cm, G3 > 2.0–3.0 cm. The positive lymph node metastases rates were 20.0%, 20.0%, and 32.6%, respectively (*P* = 0.083). The above results are summarized in Table 4.

**Table 4:** The analysis of patients of each lymph node stage for TV and GTD groups

N stage	Total	Negative (%)	Positive (%)	***P*** value	***N*** = 1 (%)	***N*** = 2 (%)	***P*** value
TV	285			0.004			0.002
V1 ≤ 0.9 cm^3^	38	32 (84.2)	6 (15.8)		3 (50)	3 (50)	
V2 0.9–3.9 cm^3^	126	98 (77.8)	28 (22.2)		12 (42.9)	16 (57.1)	
V3 3.9–14.2 cm^3^	121	74 (61.2)	47 (38.8)		15 (31.9)	32 (68.1)	
GTD				0.083			0.236
G1 ≤ 1.0 cm	5	4 (80.0)	1 (20.0)		0 (0.0)	1 (100.0)	
G2 1.0–2.0 cm	90	72 (80.0)	18 (20.0)		6 (33.3)	12 (66.7)	
G3 2.0–3.0 cm	190	128 (67.4)	62 (32.6)		24 (38.7)	38 (61.3)	

### Correlation between GTD, TV, and the metastatic lymph node stage

The study of all cases showed that there was no correlation between GTD and N stages (*P* = 0.063>0.05). No clear correlation was observed in any groups either. (*P* (G1) = 0.335, *P* (G2) = 0.667, *P* (G3) = 0.643)

The results showed that there was a company relationship between TV and N stage (*P* = 0.010). The estimated linear curve equation was as follows: *y* = 0.293 + 0.043x (*y* = N category, *x* = TV). Further analysis by groups revealed a relationship in the V2-3 group (TV> 0.9–14.2 cm^3^) (*P* = 0.020). The estimated quadratic curve equation was as follows: *y* = 0.217 + 0.201ln(x) (*y* = N category, *x* = TV). However, we did not find any correlation with the V1 group (V1 ≤ 0.9 cm^3^) (*P* = 0.945). The correlation analysis between GTD, TV, and lymph node stage is summarized in Table 5.

**Table 5:** The summary for the analysis of correlations between GTD and TV and the extent of lymph node metastases by total cases and subgroups

TV-N	***P*** value	***P*** value (total)	GTD-N	***P*** value	***P*** value (total)
≤ 0.9 cm^3^	0.945	0.010	≤ 1.0 cm	0.355	0.063
0.9–3.9 cm^3^	0.020		1.0–2.0 cm	0.667	
3.9–14.2 cm^3^	0.020		2.0–3.0 cm	0.643	

## Discussion

According to Moulla et al. who did a study in 204 patients, the tumor location was the main factor for lymph node metastases in univariate and multivariate analysis [11]. In an analysis of 566 patients, Shafazand et al. identified age less than 65 years, tumor location, and histological type (adenocarcinoma and large cell) as predictive factors for metastatic lymph nodes [12]. Another retrospective study of 379 patients by Suzuki et al. also found that the histopathological type (adenocarcinoma) was a predictive factor for mediastinal lymph node metastases [31]. In our study for NSCLCs with pulmonary nodule, in accordance with previous studies, we found that histological type (adenocarcinoma) and poor histologic differentiation are related to lymph node metastases. However, we could not identify age or tumor location as predictive factors for any lymph node metastases. The reason for this discrepancy may be due to the natural or manual differences such as the sampling process, the number of cases, and statistic methods used.

During the past several years, the relationship between GTD and lymph node metastases has been heavily studied. Yang et al. divided 198 patients into 3 GTD subgroups using 3.0 cm and 7.0 cm cutoff points. They suggested that a large GTD was related to lymph node metastases [20]. Besides, Kong et al. and Moulla et al. concluded that GTD > 3.0 cm was a predictive factor for any lymphatic metastasis [10, 11]. Furthermore, Flieder et al. found that the incidence of node metastases in patients with GTD > 2.0 cm was twice that of those with GTD ≤ 2.0 cm, suggesting that the larger tumors were accompanied with advanced disease [19]. Therefore, for NSCLC patients, tumor size is closely related to lymph node metastases.

With the advanced imaging technologies, more and more pulmonary nodules have been detected in NSCLC patients [23]. Bao et al. suggested that pathological positive lymph nodes were common in small size NSCLC patients who were diagnosed with clinical negative lymph nodes [24]. Therefore, precise preoperative diagnosis and appropriate medical methods are increasingly important for NSCLC patients. However, comparing with large tumor size, the characteristics of lymph node metastases in small size tumors are still in debate. In a study for pT1a NSCLC cases, Yu et al. classified 2268 patients into three subgroups: GTD ≤ 1.0 cm, 1.0 cm > GTD ≤ 2.0 cm, and 2.0 cm > GTD ≤ 3.0 cm. They concluded that the higher risk of lymph node involvement was accompanied with larger tumor size, which proved that tumor size is a significant predictor of lymphatic metastasis [15]. Nevertheless, according to the analysis of 185 NSCLC patients with tumors less than 2.0 cm, Shi et al. revealed that there was no lymph node metastasis in tumors < 1.0 cm, and no obvious difference was observed in the lymphatic metastatic rate between 1.6–2.0 cm and 1.0–1.5 cm tumors [13]. Moreover, Zhang et al. found that tumor size was not a reliable factor to predict lymphatic status of NSCLCs [16, 18]. In our study, our results showed that there is no significant difference for GTD with the presence or absence of lymph node metastases and there exist no obvious differences of positive metastasis rates between the three GTD groups, which indicated that GTD may not be a sensitive factor to predict positive lymph nodes.

Our study found that the TV of patients with positive lymph nodes is obviously larger than that of negative lymph node cases. This may suggest that the larger TV is associated with a higher probability of lymphatic metastasis. Besides, according to analysis by groups, we found obvious differences in metastases rates in the three groups. Several previous studies demonstrated that TV is an independent prognostic factor for NSCLC patients who underwent complete resection [21, 27, 29, 30]. Tsai et al. suggested that the TV is a more accurate indicator to evaluate tumor size as well as survival of patients with stage Ia NSCLC [32]. Furthermore, it has been proven that TV may reflect the true tumor burden better than GTD [21]. Therefore, combining with previous studies and our results, we concluded that TV is a more reliable indicator to predict if there are positive lymph nodes for tumors ≤ 3.0 cm.

According to the equation of TV and GTD mentioned above, the two TV cutoff points corresponded to 1.43 cm^3^ and 2.43 cm^3^ of GTD respectively, which were not 1.0 cm^3^ and 2.0 cm^3^ by the standard classification. In our study, the relationship between GTD and TV proved to be an exponential growth model, which means that the distribution of TV was discrete even with the same GTD category. In other words, a minor increase in GTD will lead to doubling of the TV [21]. Therefore, TV may describe the true tumor burden more accurately than GTD, especially for pulmonary nodules.

The necessary extent of lymph node dissection in the surgical treatment of NSCLC patients remains controversial [33]. Therefore, the precise preoperative staging plays a significant role in making a treatment plan. To the best of our knowledge, most previous studies mainly focused on the prediction of whether or not there are metastatic lymph nodes using GTD or TV. However, previous researches have proven that the treatment and prognosis varied a lot between N1 and N2 NSCLC patients, which means that only identifying positive lymph nodes is not enough to meet clinical needs [8]. Therefore, we evaluated the correlation between GTD and N categories to verify if there was a relationship between these two factors. The significant relationship between GTD and N stages was not observed in all the cases or in any of the groups. However, TV and N stages had a clear correlation and accord with growth models, which indicated that the larger TV may indicate higher lymph node stages. Therefore, we suggested that TV is a more sensitive marker for predicting the specific N stages in pulmonary nodule tumor masses.

According to our analysis by groups, for TV > 0.9–14.2 cm^3^, the correlation between TV and N stages is significantly obvious. Though the correlation between TV and N stage is in accordance with the growth model for all cases, we did not find any correlation when TV ≤ 0.9 cm^3^, which may be due to the limited case number of this group. According to the classification of T stage from the TNM staging system, with the larger tumor mass, the characteristics of T2, T3, and T4 have obvious differences from those of T1 since confounding factors are compromised in higher stages [8]. Several previous studies have suggested that lymph node metastases are affected by some other factors besides the tumor itself [11–13]. Therefore, we believe that the differences of characteristics in each TV interval may account for this result. Overall, based on the equation of TV and GTD mentioned before, these results revealed that TV may be an especially sensitive lymphatic predictive marker when tumor mass had a GTD> 2.0–3.0 cm. Nevertheless, for GTD ≤ 2.0 cm, both GTD and TV are not reliable factors to predict specific N stages, and further studies with larger sample sizes are needed.

With the wide use of three-dimensional reconstruction of CT and PET-CT, TV has become the other important indicator besides GTD to estimate the preoperative condition for NSCLC patients [34, 35]. Besides, its significant prognostic value has been proven for patients treated by nonsurgical means such as radiotherapy [36, 37]. Furthermore, according to our study, for NSCLC patients with pulmonary nodules, the TV value of tumor mass acquired from preoperative CT scan may provide more reliable clinical value than GTD for clinical physicians to predict nodes status, which will help physicians choose more personalized treatment projects such as surgical resection, chemotherapy, or radiotherapy for this group of patients. Therefore, considering current limited studies of the relationship between TV and lymph nodes and controversy related to the treatment for NSCLC patients with pulmonary nodules, our study may provide additional reference for predicting metastatic lymph nodes and choosing proper treatment plans to improve the prognosis for NSCLC patients with 3.0 cm or less in the greatest tumor diameter.

Several limitations of this study should be mentioned. First, this is a retrospective observational study comprised of potential biases. Second, semi-automatic volumetric measurement of solid masses surrounded by lung parenchyma was demonstrated to be reproducible with standardized protocols. Nevertheless, TV could not be calculated precisely if the tumor was accompanied with atelectasis or obstructive pneumonia. Third, since this study was carried out in a single medical center research, a larger number of cases from multiple medical centers may be needed to further evaluate our conclusion.

## Conclusions

For pulmonary nodule NSCLCs (≤ 3.0 cm), TV is a more sensitive marker for predicting positive lymph node metastases compared to GTD. The likelihood for metastasis increases with an increasing TV especially when GTD is > 2.0–3.0 cm.

## Supplementary information

**Additional file 1:Supplementary Figure 1.**

## Data Availability

The datasets generated and/or analyzed during the current study are available in the Sun Yat-sen University Cancer Center.

## Abbreviations

NSCLC: Non-small cell lung cancer
TV: Tumor volume
GTD: Greatest tumor diameter
OS: Overall survival
TNM: Tumor node metastasis
UICC: The Union for International Cancer Control
CT: Computed tomography
MRI: Magnetic resonance imaging
SPSS: Statistical package for social sciences
VATS: Video-assisted thoracoscopic surgery
GGO: Ground-glass opacity
WHO: World Health Organization
