# Supplementary figures and images for: Tumor volume is more reliable to predict nodal metastasis in non-small cell lung cancer of 3.0 cm or less in the greatest tumor diameter
Source: World J Surg Oncol. 2020 Jul 15;18:168. doi: 10.1186/s12957-020-01946-0 (PMC7364500; doi:10.1186/s12957-020-01946-0)

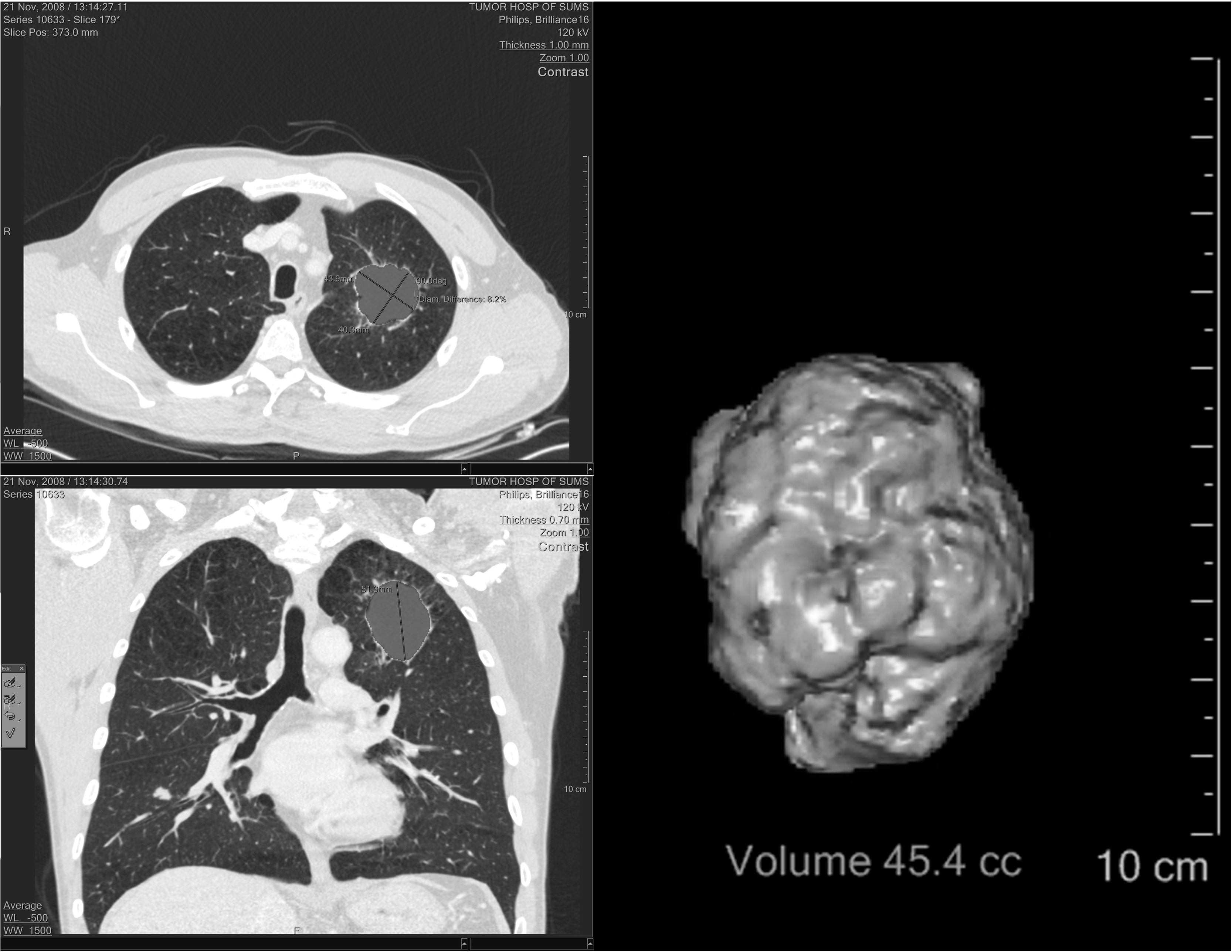

Supplement: Supplementary file 1 — Additional file 1:Supplementary Figure 1. [file 12957_2020_1946_MOESM1_ESM.tiff]
